# Structural Diversity of Hydrogen-Bonded 4-Aryl-3,5-Dimethylpyrazoles for Supramolecular Materials

**DOI:** 10.3390/ma14164550

**Published:** 2021-08-13

**Authors:** Sandra Moyano, Beatriz Diosdado, Leire San Felices, Anabel Elduque, Raquel Giménez

**Affiliations:** 1 Instituto de Nanociencia y Materiales de Aragón (INMA), Departamento de Química Orgánica, Facultad de Ciencias, CSIC-Universidad de Zaragoza, 50009 Zaragoza, Spain; sandra.moyano.perez@gmail.com; 2 Departamento de Química Inorgánica, Facultad de Ciencias, Instituto de Síntesis Química y Catálisis Homogénea (ISQCH), CSIC-Universidad de Zaragoza, 50009 Zaragoza, Spain; beadiosdado@hotmail.com; 3 Servicios Generales de Investigación SGIker, Universidad del País Vasco UPV/EHU, P.O. Box 644, 48080 Bilbao, Spain; leire.sanfelices@ehu.eus

**Keywords:** supramolecular chemistry, hydrogen bond, pyrazole, luminescence, X-ray, Hirshfeld surface analysis

## Abstract

The 1*H*-pyrazoles have high versatility and ability to form hydrogen-bonded supramolecular materials. In this study, the thermal stability, fluorescence, and H-bonding ability of the studied 3,5-dimethyl-4-(4-X-phenyl)-1*H*-pyrazoles showed large differences depending on the terminal substituent. Supramolecular structures were analyzed using X-ray diffraction and Hirshfeld surface calculations. Compounds were found to arrange in different hydrogen-bonded structures, depending on the substitution at the *para* position of the phenyl ring (X = OCH_3_, NO_2_, NH_2_). The methoxy-substituted compounds arranged in dimers through methanol bridges, the nitro-substituted compound formed supramolecular polymers or catemers, and the amino-substituted compound gave rise to a new structure based on a 2D hydrogen-bonded network.

## 1. Introduction

Hydrogen-bonded supramolecular materials are being investigated in diverse research areas such as for drug development [1], energetic materials [2], porous supramolecular organic frameworks [3], or functional soft materials [4,5]. By making use of engineered supramolecular interactions, such as directional hydrogen bonds, small predesigned molecular units can generate structural complexity and improve functions in organic materials, avoiding the use of complex covalent synthetic routes.

The particular structure of 1*H*-pyrazole makes this cycle a versatile supramolecular synthon for crystal engineering [6,7]. The different characteristics of the two adjacent nitrogens in the five-member cycle of 1*H*-pyrazole, namely the pyrrol-type nitrogen (N1-H) and the pyridine-type nitrogen (N2), allow hydrogen-bonded interactions to be established with different topologies (monodentate, exobidentate) [8,9], as well as multiple bridge modes to be established between pyrazole cycles such as dimers, trimers, tetramers, or polymeric structures (catemers) [10,11]. Such structural diversity and the dynamic character of the hydrogen bonds mean pyrazole derivatives have great potential for the development of functional materials.

Substitution of the 1*H*-pyrazole cycle at the 4 position with a phenyl ring increases the anisotropy and endows the structure with fluorescent properties. In particular, the 3,5-dimethyl-4-arylpyrazole platform has been incorporated in different structures, yielding functional soft materials such as liquid crystals [12,13] or responsive supergels [14], in which hydrogen bonding plays an important role in their organization and properties. Also of interest is the ability to maintain fluorescence in the solid or aggregated state and show aggregation-induced emission (AIE) [12,14]; that is, the luminescence is not quenched but enhanced at high concentrations or in the bulk [15,16], contrary to the behavior of standard organic fluorophores.

**Scheme 1:**
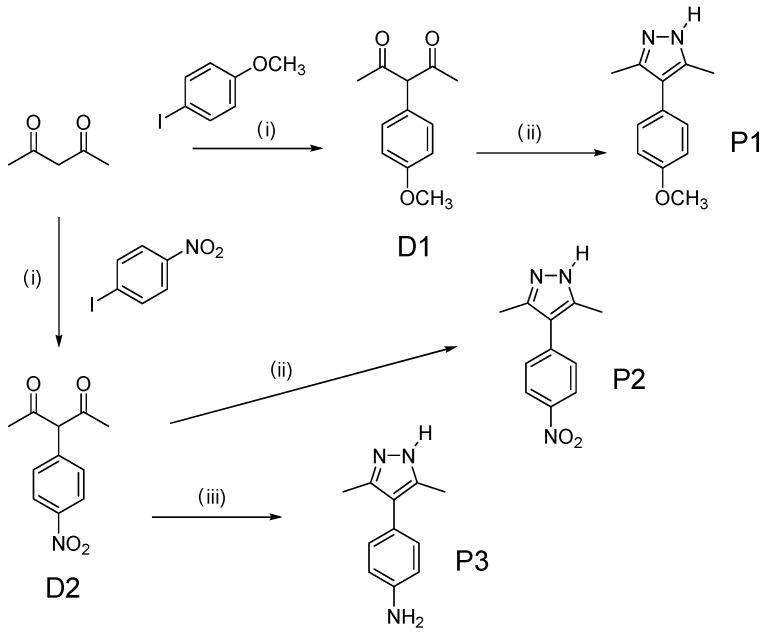
Synthetic routes for the preparation of the final pyrazole compounds: (i) (1) CuI, L-proline, K_2_CO_3_, DMSO; (2) HCl 1M; (ii) N_2_H_4_·H_2_O, EtOH; (iii) N_2_H_4_·H_2_O, graphite, EtOH.

In order to shed light on the structure and hydrogen bonding modes of anisotropic 3,5-dimethyl-4-arylpyrazoles that would help to develop predictable supramolecular organizations, selected model structures with nitrogen- or oxygen-derived substituents at the *para* position of the phenyl ring were studied here in the solid state. Three compounds derived from 3,5-dimethyl-4-(4-X-phenyl)-1*H*-pyrazoles—namely X methoxy, nitro, and amino groups (Scheme 1)—were synthesized, their thermal and optical properties were studied, and their supramolecular structures were analysed using X-ray diffraction and Hirshfeld surface calculations.

## 2. Materials and Methods

### 2.1. Techniques

All starting materials and solvents were obtained from commercial sources and used without purification. Nuclear magnetic resonance (NMR) spectra were measured on a Bruker Avance 400 spectrometer. Chemical shifts are given in ppm relative to tetramethylsilane (TMS) and the solvent residual peak was used as the internal standard. Microanalyses were performed with a Perkin-Elmer 2400 microanalyzer. Infrared (IR) spectra were recorded on a Nicolet Avatar FTIR spectrophotometer using KBr pellets. Mass spectra (MS) were obtained on a MICROFLEX Bruker spectrometer. Optical microscopy was studied using an Olympus BX51 microscope equipped with a Linkam THMS600 hot stage. Thermogravimetric analysis (TGA) was performed using a TA Instruments Q5000 apparatus at a heating rate of 10 °C min^−1^ under a nitrogen atmosphere. Transition temperatures and melting enthalpies were obtained by differential scanning calorimetry (DSC) using a TA Instruments Q20 device at a rate of 10 °C min^−1^. Optical absorption spectra were recorded with a UV4-200 UV-Vis spectrophotometer from ATI Unicam. Fluorescence spectra were recorded with a Perkin-Elmer LS50B system. Thin-film spectra were recorded by front-face detection.

### 2.2. Synthetic Methods (See Scheme 1)

#### 2.2.1. Synthesis of 3-(4′-Methoxyphenyl)Pentane-2,4-Dione (**D1**)

A suspension of 4-iodomethoxybenzene (19.4 mmol, 4.54 g), K_2_CO_3_ (97.1 mmol, 13.42 g), CuI (1.9 mmol, 0.36 g), and (L)-Proline (3.9 mmol, 0.45 g) in DMSO (40 mL) was stirred under argon for 5 min and then acetylacetone (58.2 mmol, 5.82 g) was added to the mixture. After three argon–vacuum cycles, the reaction mixture was stirred at 70 °C for 18 h. Once the reaction had finished, the mixture was poured over an aqueous solution of HCl 1M (150 mL). After this, the product was extracted with ethyl acetate and washed with water. The organic layer was dried with MgSO_4_, filtered, then the solution was evaporated. The obtained product was purified by column chromatography (silica gel, hexane/ethyl acetate, 15:1) to give a yellow oil (yield: 1.6 g, 41%; ^1^H NMR (400 MHz, CDCl_3_) δ/ppm (enol tautomer) 16.63 (s, 1H), 7.08–7.06 (m, 2H), 6.92–6.90 (m, 2H), 3.83 (s, 3H), 1.88 (s, 6H). ^13^C NMR (100 MHz, CDCl_3_): δ/ppm 191.2, 158.9, 132.1, 129.1, 114.6, 114.1, 55.2, 24.1).

#### 2.2.2. Synthesis of 3,5-Dimethyl-4-(4′-Methoxyphenyl)-1*H*-Pyrazole (**P1**)

Hydrazine hydrate (7.2 mmol, 0.36 mg) was added to a mixture of **D1** (5.8 mmol, 1.2 g) in ethanol (10 mL) at room temperature. Then, the mixture was heated under reflux for 2 h. The reaction mixture was evaporated under reduced pressure and the residue was purified in ethanol to give a white solid (yield: 1.1 g, 91 %; ^1^H NMR (400 MHz, CDCl_3_) δ/ppm 7.22–7.19 (m, 2H), 6.98–6.95 (m, 2H), 3.85 (s, 3H), 2.30 (s, 6H). ^13^C NMR (100 MHz, CDCl_3_): δ/ppm 158.0, 141.7, 130.4, 126.0, 118.0, 113.9, 55.2, 11.5. MS (MALDI+, dithranol) *m/z*: 203.1 [M + H]+. Analysis found C, 70.91; H, 6.94; N, 13.83. Calc for [C_12_H_14_N_2_O]: C, 71.26; H, 6.98; N, 13.85%. FTIR ν/cm^−1^ 3175 (N-H), 1613, 1577, 1530, 1247 (C-O-C)).

#### 2.2.3. Synthesis of 3-(4′-Nitrophenyl)Pentane-2,4-Dione (**D2**)

A solution of 4-nitroiodobenzene (11.5 mmol, 2.86 g), K_2_CO_3_ (57.2 mmol, 7.90 g), CuI (1.14 mmol, 0.22 g), and (L)-Proline (2.28 mmol, 0.26 g) in DMSO (30 mL) was stirred under argon for 5 min, then acetylacetone (34.3 mmol, 3.43 g) was added to the mixture. After three argon–vacuum cycles, the reaction mixture was stirred at 60 °C for 24 h. Once the reaction had finished, the mixture was poured over an aqueous solution of HCl 1M (100 mL). After this, the product was extracted with ethyl acetate and washed with water. The organic layer was dried with MgSO_4_, filtered, and then the solution was evaporated. The obtained product was purified by column chromatography (silica gel, hexane/ethyl acetate, 15:1) to give a yellow oil (yield: 1.9 g, 74%; ^1^H NMR (400 MHz, CDCl_3_) δ/ppm (enol tautomer) 16.75 (s, 1H), 8.26–8.24 (m, 2H), 7.40–7.38 (m, 2H), 1.89 (s, 6H). ^13^C NMR (100 MHz, CDCl_3_): δ/ppm 190.4, 147.3, 144.0, 132.2, 124.0, 113.6, 24.1).

#### 2.2.4. Synthesis of 3,5-Dimethyl-4-(4′-Nitrophenyl)-1*H*-Pyrazole (**P2**)

Hydrazine hydrate (1.7 mmol, 0.08 mg) was added to a mixture of **D2** (1.4 mmol, 0.3 g) in ethanol (10 mL) at room temperature. Then, the mixture was heated under reflux for 2 h. The reaction mixture was evaporated under reduced pressure and the residue was purified by recrystallization in ethanol to give a white solid (yield: 230 mg, 76%; ^1^H NMR (400 MHz, CDCl_3_) δ/ppm 8.29–8.27 (m, 2H), 7.45–7.42 (m, 2H), 2.35 (s, 6H). ^13^C NMR (100 MHz, CDCl_3_): δ/ppm 141.0, 135.6, 129.5, 123.8, 11.8. MS (MALDI+, dithranol) *m*/*z*: 218.0 [M + H]+. Anal. Found C, 60.40; H, 5.00; N, 19.21. Calc for [C_11_H_11_N_3_O_2_]: C, 60.82; H, 5.10; N, 19.34%. FTIR ν/cm^−1^ 3174 (N-H), 1596, 1515 (NO_2_), 1342 (NO_2_)).

#### 2.2.5. Synthesis of 3,5-Dimethyl-4-(4′-Aminophenyl)-1*H*-Pyrazole (**P3**)

Graphite (2.3 g) was added to a solution of **D2** (4.50 mmol, 1 g) in ethanol (50 mL) and stirred under an Ar atmosphere. Then, hydrazine hydrate (36.2 mmol, 1.81 g) was added. The mixture was stirred under reflux for 12 h. Then, the mixture was filtered over Celite^®^ to remove graphite and washed with hot ethanol. The solvent was evaporated and the product was purified by recrystallization in water to give a white solid (yield: 618 mg, 72%; ^1^H NMR (400 MHz, CDCl_3_) δ/ppm 7.07–7.05 (m, 2H), 6.75–6.73 (m, 2H), 3.78 (s, 2H), 2.27 (s, 6H). ^13^C NMR (100 MHz, CDCl_3_): δ/ppm 144.7, 130.2, 123.7, 118.4, 115.2, 11.5. MS (MALDI+, dithranol) *m*/*z*: 188.2 [M + H]+. Anal. Found C, 70.63; H, 7.11; N, 22.30. Calc for [C_11_H_13_N_3_]: C, 70.56; H, 7.00; N, 22.44%. FTIR ν/cm^−1^ 3401 (NH_2_), 3290 (NH_2_), 3177 (N-H), 1618, 1585, 1537).

### 2.3. Single-Crystal Preparation and Measurement by X-ray Diffraction

Single crystals of **P1** could only be obtained as a methanol solvate **P1·CH_3_OH**. Crystals were grown by vapor diffusion of methanol into a solution of the compound in dichloromethane at room temperature. They were fragile and quickly set up in the instrument at 150 K to prevent loss of the solvent and degradation. X-ray diffraction data for **P1·CH_3_OH** were measured at 150 K on an Xcalibur S diffractometer from Oxford Diffraction Ltd., using Mo-Kα radiation (λ = 0.71073 Å). The structure was solved with SIR-92. All hydrogen atoms were placed geometrically except H1N, H3O, H4N, and H4O, which were localized in electronic density maps and refined with the ShelXL refinement package. Single crystals of **P2** and **P3** were obtained by vapor diffusion of hexane into a dichloromethane solution at 5 °C and were stable after filtering and drying at room temperature. Single-crystal X-ray diffraction data for **P2** and **P3** were acquired at 100 K using a single-source Agilent SuperNova Atlas diffractometer using Cu-Kα radiation (λ = 1.54184 Å). The structure was solved with the ShelXT structure solution program using intrinsic phasing and refined with the ShelXL refinement package using least squares minimization. A summary of the crystal data and refinement parameters is given in Table 1.

## 3. Results and Discussion

### 3.1. Synthesis and Characterization

Pyrazole compounds were obtained following the synthetic route depicted in Scheme 1. The precursory 1,3-diketones were synthesized through a copper-catalyzed arylation reaction of acetylacetone with *p*-iodomethoxybenzene or *p*-iodonitrobenzene [12]; details are given in Materials and Methods. **P1** and **P2** were obtained by reaction of diketones **D1** and **D2** with hydrazine hydrate, respectively. **P3** was obtained in high yield from the nitro-substituted acetylacetone **D2** in just one step, through a double reduction method using graphite and hydrazine hydrate. Pyrazole compounds were characterized by ^1^H-NMR and ^13^C-NMR. Elemental analysis confirmed the stoichiometry and purity of the compounds. In addition, mass spectra in the form of the protonated [M + H]^+^ signal was obtained for all cases.

The IR spectra of the as-obtained solids gave preliminary proof of the existence of supramolecular structures by hydrogen bonding. IR spectra of **P1** and **P2** showed bands corresponding to the N-H stretching of the pyrazole ring in the associated region (3174 or 3175 cm^−1^). The IR spectrum of **P3** showed different N-H bands at 3401, 3290, and 3177 cm^−1^, indicating a complex arrangement involving H-bonding in both amino and pyrazole N-H groups.

### 3.2. Thermal Properties

**Figure 1:**
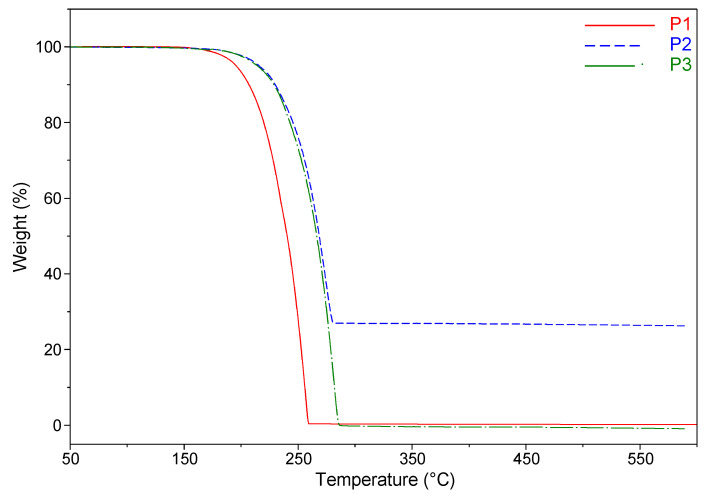
Thermogravimetric analysis curves of the studied pyrazoles.

The thermal properties of the as-obtained pyrazoles were analyzed by optical microscopy, thermogravimetry (TGA), and differential scanning calorimetry (DSC) (Table 2). **P1** does not melt to an isotropic liquid on heating but sublimates at atmospheric pressure. Consistently, the thermogravimetric analysis under an inert atmosphere showed a starting temperature for weight loss of 153 °C, a 5 % weight loss at 196 °C, and quantitative weight loss at 259 °C in a ramp experiment (Figure 1). **P2** and **P3** showed higher thermal stability and melted into isotropic liquids at higher temperatures than **P1**. As for the TGA experiment for **P2** and **P3,** both melted before the weight loss started and showed similar decomposition curves, although **P2** did not decompose without complete volatile emission.

### 3.3. Optical Properties

**Figure 2:**
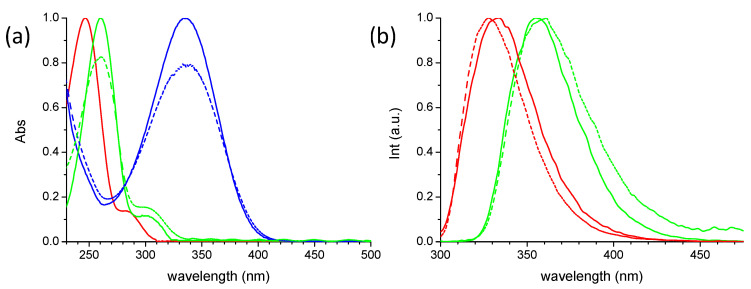
(**a**) UV-Vis absorption spectra and (**b**) fluorescence spectra for **P1** (red), **P2** (blue), and **P3** (green). Data relate to samples studied in solution (solid lines) and in film (dashed lines).

UV-Vis absorption and emission spectra were studied in tetrahydrofuran (THF)-diluted solution and in film state (Table 3). **P1** and **P3** display two absorption bands in the UV region corresponding to π-π * and n-π * transitions. **P3** shows a bathochromic shift with respect to **P1** corresponding to the electronic effects of the amino group vs. the methoxy group. **P2** displays one absorption band at higher wavelengths corresponding to a charge transfer band due to the effect of the acceptor nitro group and the weak donor effect of the 4-pyrazole group (Figure 2a). **P1** and **P3** are fluorescent in the near UV region in THF solution and show quite similar emission bands in thin film (Figure 2b). In contrast, **P2** does not show fluorescence. This behavior is common for nitroaromatic compounds due to the existence of efficient non-radiative decay processes in solution and the influence of secondary interactions of the nitro group in crystals (see below) [17].

### 3.4. Single-Crystal Structures and Supramolecular Packing

In order to study the supramolecular hydrogen-bonded abilities of these 1*H*-pyrazoles, their single-crystal structures were solved. **P1** was obtained as a methanol solvate, while **P2** and **P3** crystallized were obtained as pure compounds.

#### 3.4.1. Intramolecular Parameters

**Figure 3:**
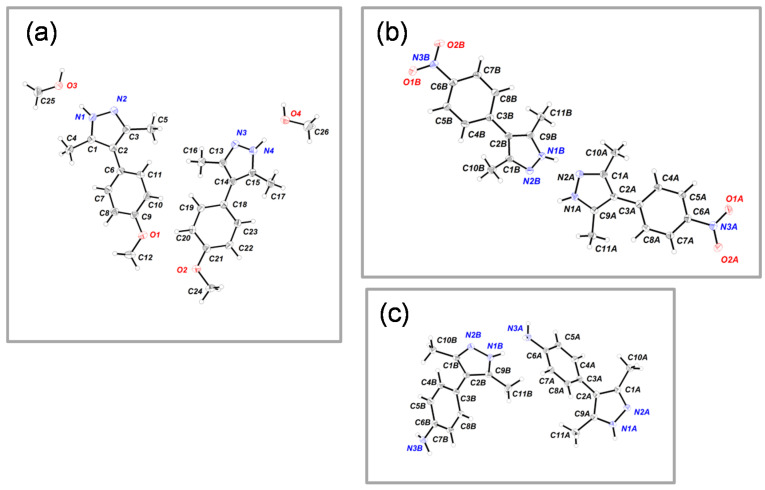
ORTEP diagram (50% probability of displacement ellipsoids) for various structures: (**a**) **P1·CH_3_OH**; (**b**) **P2**; (**c**) **P3**.

Concerning their crystallographic information, **P1·CH_3_OH** crystallized in the triclinic system (space group P¯ī) with four molecules of **P1** and four methanol molecules in the unit cell (Z = 4). The asymmetric unit is formed by two crystallographically independent molecules and two molecules of methanol. (Figure 3a) **P2** crystallized in the monoclinic system (space group P2_1_/c) with Z = 8 and two crystallographically independent molecules (Figure 3b). **P3** also crystallized in the monoclinic system (space group C2/c) with Z = 16 and two crystallographically independent molecules (Figure 3c).

At the molecular level, the distances and bond lengths are very similar for the three structures (Table 4) and correspond to the expected values for pyrazole and benzene cycles. Typical measurements for pyrazole rings with alternating distances were found, namely carbon–carbon distances in the pyrazole ring d(pz^3^-pz^4^) larger than d(pz^4^-pz^5^), which correspond to a double-unsaturated ring. Bond distances between pyrazole and benzene rings d(pz^4^-Ph) were around 1.46–1.48 Å, indicating low conjugation. Molecules were not planar but were significantly twisted, with pyrazole and benzene forming at angles **α** of 42° for **P1**, 44 or 46° for **P3**, and 34 or 37° for **P2**. Furthermore, **P2** was less twisted and the nitro substituent was coplanar with the phenyl ring.

A search of the literature provided only three more single-crystal structures containing the skeleton of 3,5-dimethyl-4-(4-X-phenyl)-1*H*-pyrazole (X = O, N). They were the structures with CSD reference codes ZUWLEI (X = OH) [18], OKIKAW (substituted at the 4 position of the pyrazole with a 3,4,5-trimethoxyphenyl group) [13]*,* and REYYIF (X = NHCOPh) [12]. In addition, one N-substituted pyrazole GIQZAK (3,5-dimethyl-4-(2-bromo-4-methoxy-5-hydroxyphenyl)-*N*-phenylpyrazole) was found [19]. For all three 1*H*-pyrazole structures, the phenyl and pyrazole cycles are not coplanar, with angles laying in the same range as found here (44.9°, 46.7°, and 45.6° or 36.0°, respectively). Non-planar structures were also found for other 3,5-dimethyl-4-aryl-1*H*-pyrazoles, such as NUBZUH (X = COOH) [20], as well as two bispyrazoles NILREH (aryl = 2-(3,5-dimethylpyrazol-4-yl)phenyl) [21] and BOTBUJ (aryl = 3-(3,5-dimethylpyrazol-4-yl)phenyl) [22] and the pyrazolium salts RABNEN (CF_3_COO^−^, X = COOH) [23], NUCBAQ (NO_3_^−^, X = COOH) [20], and SUGTIA (Cl^−^, X = PO_3_H_2_) [24]. In contrast, a planar geometry was found in the single-crystal structure of 4-phenyl-1*H*-pyrazole, which provided evidence of the influence of the 3,5-dimethyl substitution [25].

#### 3.4.2. Intermolecular Hydrogen Bonds and Packing

**Figure 4:**
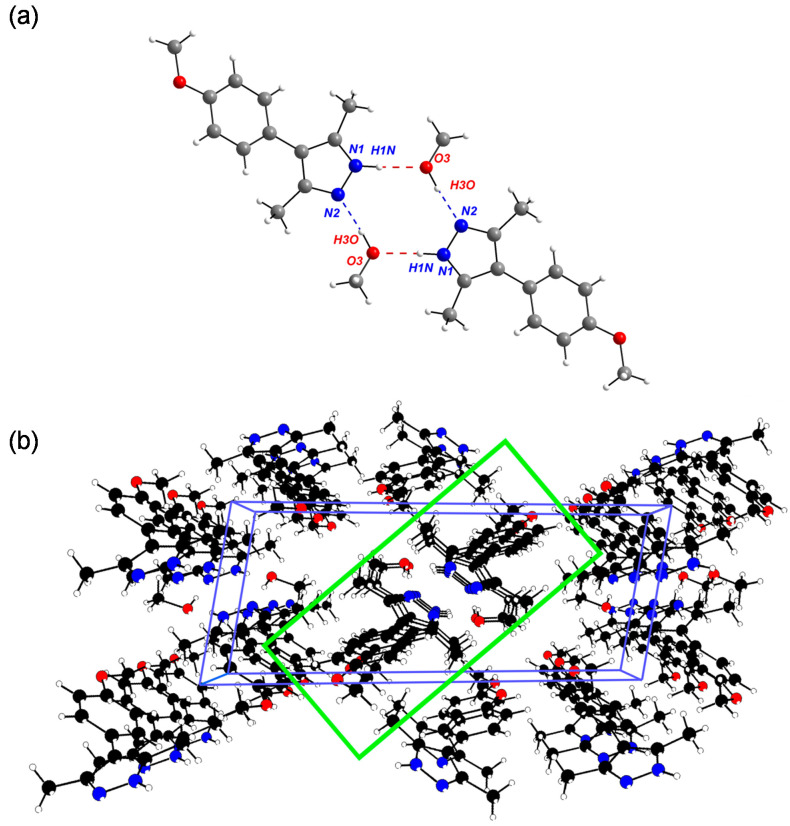
(**a**) Hydrogen bond formation in **P1·CH_3_OH**. (**b**) Packing of **P1·CH_3_OH** along the *b* axis. A pile of dimers is highlighted with a green rectangle.

Concerning the formation of hydrogen bonds, each independent molecule of **P1** interacts with two molecules of methanol through hydrogen bonds. These two molecules of methanol interact with another pyrazole in such a way that two pyrazoles form a dimer through methanol bridges, as shown in Figure 4a. The hydrogen atoms H1N or H4N of the donating pyrazoles interact with the oxygen acceptor of methanol (O3 or O4) at distances of 1.863 Å or 1.828 Å, respectively. The acceptor nitrogen atoms N2 and N3 interact with the hydrogen atoms of methanol (H3O and H4O) at distances of 1.79 Å and 1.864 Å, respectively. The donor–acceptor distances are around 2.77 Å and the angles deviate slightly from linearity (175°). All data are shown in Table 5. This supramolecular structure stacks along the *b* axis (Figure 4b).

**Figure 5:**
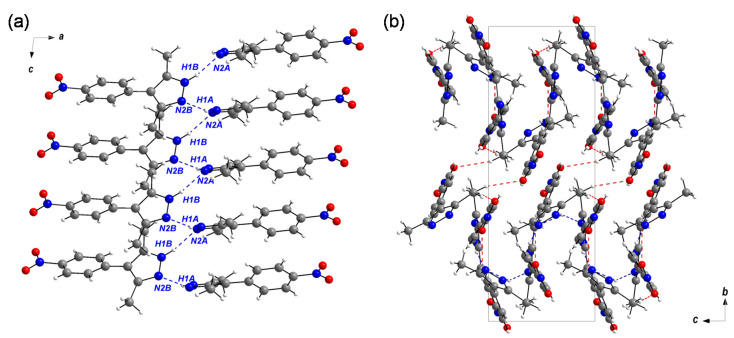
(**a**) Packing of **P2** in the *ac* plane, detailing the supramolecular polymer formed by the hydrogen bonds between pyrazole cycles (blue broken lines). (**b**) View of the *bc* plane with details of the interactions involving the nitro group (red broken lines).

The crystalline packing of **P2** was different to **P1**. In this case, the pyrazole rings were chained, forming a supramolecular polymer through hydrogen bonds along the *c* axis (Figure 5a, blue broken lines) involving the two crystallographically independent molecules A and B alternatively. This type of polymeric structure is known as a catemer. Pyrazole rings are twisted at 60.3° in a zig-zag manner in order to be able to form the chain. The polymers have their nitrophenyl substituents interdigitated with parallel catemers and there are additional hydrogen contacts between these catemers, involving the oxygens of the nitro substituents of one catemer and the methyl groups and CH of the phenyl groups of adjacent chains (C4B-H4B···O2A, C10A-H10A···O2B, C11B-H11B···O1A) (Table 6) (Figure 5b, red broken lines).

**Figure 6:**
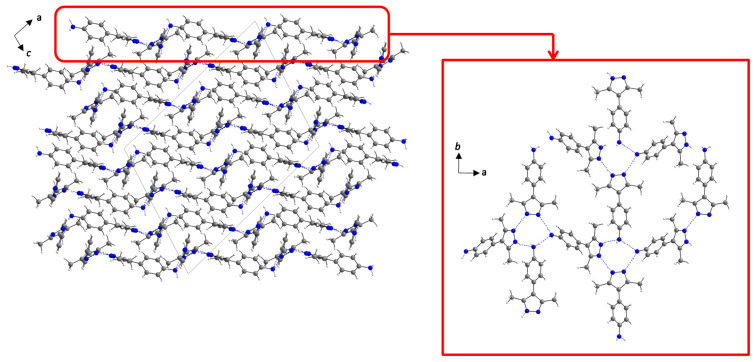
Packing of **P3** in the *ac* plane and supramolecular 2D network formed by hydrogen bonds between four molecules formed in the *ab* plane.

**P3** show very different packing to **P1** and **P2**, as a consequence of the H-bonding donating ability of the amino substituent. In this case, the packing consists of a 2D network of hydrogen bonds in the *ab* plane. Each molecule is engaged in hydrogen bonds with the other three molecules, giving rise to the formation of supramolecular cycles involving 6 nitrogen atoms, in which two molecules interact through the pyrazole ring and the other two interact through the amino group (Figure 6, Table 7).

A comparison of the structures points out that the supramolecular organization is highly dependent on the substitution of the aromatic ring and on the use of methanol in the crystallization process, giving rise to different supramolecular motives, some of which are newly reported here. Methanol solvates are observed when this solvent is used in the crystallization process to bridge two pyrazole molecules via hydrogen bonding. The same result was previously reported for a methanol solvate [12]. Unfortunately, good quality crystals of **P1** could not be grown in the absence of methanol, which prevented the study of its non-solvated form. The catemer structure found for **P2** is unprecedented for neutral 3,5-dimethyl-4-(4-X-phenyl)pyrazoles, as other previously reported substitutions did not give rise to pyrazole–pyrazole interactions, but rather a head-to-tail arrangement of pyrazole-forming chains [13,19,20]. In **P2**, the pyrazole–pyrazole polymeric chains run parallel and are stabilized by additional secondary interactions involving the nitro group. Finally, a new motif based on supramolecular mixed cycles that form a 2D network was found for the amino substituent compound **P3** due to the participation of both amino and pyrazole groups.

#### 3.4.3. Hirshfeld Surface Analysis

**Figure 7:**
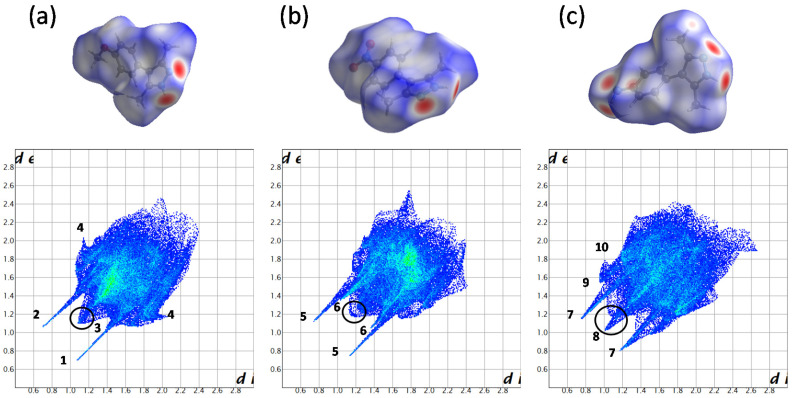
Hirshfeld surfaces mapped over the d_norm_ and 2D fingerprint plots for (**a**) **P1•CH_3_OH**, (**b**) **P2**, and (**c**) **P3.**

Hirshfeld surface analysis was performed in order to illustrate the different intermolecular interactions, and in particular hydrogen bonding. Hirshfeld surfaces mapped over the d_norm_ and 2D fingerprint plots of the three crystal structures (Figure 7) were calculated with the CrystalExplorer 17.5 program [26]. On these surfaces, the short and dominant interactions are displayed as red areas indicating the presence of close contacts such as hydrogen bonds.

The fingerprint plot for **P1•CH_3_OH** shows two sharp spikes represented by regions 1 and 2 in Figure 7a, which correspond to quite strong hydrogen bond interactions between pyrazole and methanol molecules (1: N···HO and 2: NH···O), as reflected in the parameters listed in Table 5, contributing nearly 20% of the total surface area. In addition, short O···H contacts involving the methoxy group (region 3) and C···H contacts involving methyl groups (region 4) can be seen.

The 2D plot for **P2** shows two clear spikes corresponding to the hydrogen bond interactions between pyrazoles (N-H) (regions labeled as 5 in Figure 7b), representing 15.3% of the surface area. the important contribution in the crystal stabilization of N-O..H-C interactions (24.7 %) (shortest contact region labeled as 6) involving the nitro group can be observed, as listed in Table 6.

For **P3**, the two regions labeled as 7 (Figure 7c) represent N···H hydrogen bonds involving both pyrazole and amino groups, contributing 17.5% of the total area. The thicker aspects of these spikes compared to previous compounds indicate that more hydrogen bonds are present, although they are at larger di + de, suggesting weaker hydrogen bonds. Area 8 is assigned to short H···H contacts between methyl groups of two neighboring pyrazoles (see red spot on the surface of Figure 7c). Areas 9 and 10 indicate the existence of Car-H and N-H, respectively.

It is interesting to note that although the molecular structures and the supramolecular structures are different (dimers, catemers, or 2D networks), the hydrogen bonds contribute to the stabilization of the structure in quite similar proportions (20%, 15.3%, and 17.5%, respectively). In all cases, the H···H intermolecular contacts (van der Waals forces), in the middle region of the plot with minimum values marked with a circle make a major contribution to the crystal packing. Similar values can be observed for **P1•CH_3_OH** and **P3** (60.3% and 58.3%, respectively), with lower occurring for **P2** (36.1%) due to the existence of the additional O···H interactions.

## 4. Conclusions

The thermal stability, fluorescence, and H-bonding ability of the studied 5-dimethyl-4-(4-X-phenyl)-1*H*-pyrazoles showed clear differences depending on the terminal substituent. From a comparison of the supramolecular structures it was observed that the hydrogen bonds between 1*H*-pyrazole cycles are greatly influenced by the possibility of forming additional interactions with the chemical group at the para position of the phenyl ring. The nitro substituent, with only hydrogen bond acceptor ability, allows the pyrazole–pyrazole interactions, yielding a catemer or supramolecular polymeric chain. To this structure there is an important contribution of additional short contacts involving the nitro group, which is also reflected in the thermal properties. In contrast, the amino group, which has donor characteristics, interacts with the pyrazole ring and participates in the formation of supramolecular mixed cycles formed by four molecules in the form of an amino–amino–pyrazole–pyrazole structure, giving rise to 2D supramolecular sheets. Hydrogen bonding in the 2D network is weaker than in the dimer or catemer configuration. In addition, the use of protic solvents in the crystallization process as methanol has a fundamental importance as it participates in the formation of hydrogen bonds, bridging two pyrazoles and giving rise to closed “dimers” instead of catemer structures.

## Figures and Tables

**Table 1:** Crystal data resolution and refinement parameters.

	P1·CH_3_OH	P2	P3
Chemical formula	C_13_H_18_N_2_O_2_	C_11_H_11_N_3_O_2_	C_11_H_13_N_3_
Formula mass	234.29	217.23	187.24
Crystal system	Triclinic	Monoclinic	Monoclinic
*a* (Å)	8.0633 (8)	14.5887 (2)	21.6136 (2)
*b* (Å)	8.2470 (6)	19.8458 (2)	11.73111 (12)
*c* (Å)	19.7880 (18)	7.24361 (12)	16.6400 (2)
*α* (°)	80.980 (7)	90	90
*β* (°)	79.977 (8)	97.5482 (15)	107.1683 (13)
*γ* (°)	88.969 (7)	90	90
Unit cell volume (Å^3^)	1279.7 (2)	2079.02 (5)	4031.10 (8)
Temperature (K)	150 (1)	99.97 (11)	99.98 (10)
Space group	*P*¯ī	*P*2_1_/c	*C*2/c
N° of formula units per unit cell, *Z*	4	8	16
Radiation type	MoKα	CuKα	CuKα
Absorption coefficient, *μ* (mm^−1^)	0.083	0.818	0.603
N° of reflections measured	8328	15154	15405
N° of independent reflections	4503	3876	3739
*R*_int_	0.0337	0.0285	0.0246
Final *R*_1_ values (*I* > 2*σ*(*I*))	0.0572	0.0332	0.0375
Final w*R*(*F*^2^) values (*I* > 2*σ*(*I*))	0.1060	0.0824	0.0993
Final *R*_1_ values (all data)	0.0942	0.0386	0.0415
Final w*R*(*F*^2^) values (all data)	0.1197	0.0866	0.1029
Goodness of fit on *F*^2^	1.081	1.044	1.057

**Table 2:** Thermal properties.

Compound	Phase Transition ^1^ T (°C) (∆H (kJ mol^−1^))	T_5%_ (°C) ^2^
**P1**	Cr 153 ^3^ V	196
**P2**	Cr 193 (24.2) I	216
**P3**	Cr 171 (27.4) I	214

^1^ From DSC thermograms, the first heating cycle at 10 °C min^−1^. Cr: crystal; V: vapor; I: isotropic liquid. ^2^ Temperature of 5% weight loss, heating rate 10 °C min^−1^. ^3^ Data obtained by optical microscopy using a hot stage.

**Table 3:** UV-Vis absorption and fluorescence data.

Compound	λ_abs_ (THF) (nm)	ε (L mol^−1^ cm^−1^)	λ_em_ (THF) (nm)	λ_abs_ (Film) (nm)	λ_em_ (Film) (nm)
**P1**	247, 282	1.41 × 10^4^, 1.96 × 10^3^	333	250, 289	328
**P2**	337	1.14 × 10^4^	-	333	-
**P3**	260, 297	1.68 × 10^4^, 1.87 × 10^3^	355	261, 295	361

**Table 4:** Comparison of representative distances d (Å) and torsion angles between the pyrazole and the phenyl ring α (°).

Compound	d(pz^3^-pz^4^)	d(pz^4^-pz^5^)	d(pz^4^-Ph)	α
**P1·CH_3_OH**	1.411	1.390	1.480	42.3
	1.417	1.389	1.480	42.4
**P2**	1.418	1.396	1.464	36.7
	1.424	1.393	1.466	34.3
**P3**	1.419	1.387	1.475	44.2
	1.417	1.392	1.477	45.7

**Table 5:** Intermolecular H-bond data for **P1·MeOH.**

D-H···A	D-H (Å)	H···A (Å)	D···A (Å)	DH···A (°)
N1-H1N···O3	0.91	1.863	2.769	175
N4-H4N···O4	0.95	1.828	2.777	174
O3-H3O···N2 ^1^	0.95	1.790	2.739	176
O4-H4O···N3 ^2^	0.91	1.864	2.768	176

Note: ^1^ = (x + 1, −y + 3, −z); ^2^ = (x + 1, −y + 2, −z + 1).

**Table 6:** Intermolecular H-bond data and nitro contacts for **P2.**

D-H···A	D-H (Å)	H···A (Å)	D···A (Å)	D-H···A (°)
N1A-H1A···N2B ^1^	0.88	2.02	2.874	162
N1B-H1B···N2A ^2^	0.88	2.02	2.863	160
C4B-H4B···O2A ^3^	0.95	2.57	3.458	155
C10A-H10A···O2B ^4^	0.98	2.54	3.488	164
C11B-H11B···O1A	0.98	2.51	3.485	171

Note: ^1^ = (−1 + x, 1/2 − y, −1/2 + z); ^2^ = (1 + x, y, z); ^3^ = (x, 1/2 − y, 1/2 + z); ^4^ = (−x, −y, 1 − z).

**Table 7:** Intermolecular H-bond data for **P3.**

D-H···A	D-H (Å)	H···A (Å)	D···A (Å)	D-H···A (°)
N1A-H1A···N2B ^1^	0.88	2.10	2.915	153
N1B-H1B···N3A ^2^	0.88	2.04	2.906	166
N3A-H3AB···N3B ^3^	0.90	2.18	3.075	171
N3B-H3BA···N2A ^4^	0.90	2.16	3.047	167

Note: ^1^ = (x, 1 − y, 1/2 + z); ^2^ = (x, −y, 1/2 + z); ^3^ = (−1/2 + x, −1/2 + y, z); ^4^ = (1/2 + x, −1/2 + y, z).

## Data Availability

The CCDC numbers 2073139, 2073145, and 2073146 contain the supplementary crystallographic data for the pyrazole compounds studied in this work, **P1•CH_3_OH**, **P2** and **P3**, respectively. These data can be obtained free of charge from the Cambridge Crystallographic Data Centre via http://www.ccdc.cam.ac.uk/data_request/cif.
